# FotoFinder Dermoscopy Analysis and Histopathological Correlation in Primary Localized Cutaneous Amyloidosis

**DOI:** 10.5826/dpc.1103a57

**Published:** 2021-05-20

**Authors:** Mahajabeen S Madarkar, Varsha R Koti

**Affiliations:** Department of Dermatology, S N Medical College, Bagalkot, Karnataka, India

**Keywords:** macular amyloidosis, lichen amyloidosis, FotoFinder dermoscopy, white rosettes

## Abstract

**Background:**

Primary localized cutaneous amyloidosis (PLCA) causes extracellular proteinaceous deposits in skin. It is clinically divided into macular amyloidosis, lichen amyloidosis and nodular amyloidosis. Atypical presentations of PLCA make the diagnosis challenging, requiring biopsy to confirm amyloid deposition in the upper papillary dermis.

**Objectives:**

This study used FotoFinder dermoscopy to characterize lichen and macular amyloidosis and correlated the dermoscopic features with histopathological findings.

**Methods:**

This cross-sectional study enrolled patients with a clinical and histopathological diagnosis of PLCA. Dermoscopic examination was performed using the FotoFinder dermoscope, which provides a range of magnification from 20× to 140×.

**Results:**

A total of 30 patients were included in the study. Common dermoscopic patterns of MA were white or brown central hubs, and common patterns of LA were white structureless, scar-like areas and central hubs. New dermoscopic findings were a day lily appearance in MA and white rosettes in LA.

**Conclusions:**

Dermoscopy plays a pivotal role in demonstrating characteristic findings of PLCA. These findings were well corelated with histopathology, thus avoiding unnecessary biopsy for arriving at an accurate diagnosis of PLCA.

## Introduction

The term “amyloid” means a starch-like substance. It refers to extracellular proteinaceous deposits that develop from the aggregation of misfolded plasma proteins and that are resistant to proteolytic digestion. Amyloid has distinctive physical properties. Amyloid proteins have an antiparallel beta sheet conformation and form non-branching linear fibrils.

Amyloidosis is differentiated into localized cutaneous amyloidosis and cutaneous amyloidosis due to systemic disease. Primary localized cutaneous amyloidosis (PLCA) is clinically divided into the subtypes macular amyloidosis (MA), lichen (or papular) amyloidosis (LA), and nodular amyloidosis [1]. LA is the most common form seen in Asia [2], South America and Europe. The etiology is unknown [3]. In PLCA, there is deposition of amyloid in previously normal skin with no evidence of internal organ deposition. With atypical presentations of PLCA the diagnosis is challenging, so skin biopsy becomes inevitable to confirm the presence of amyloid in the upper dermal papillae.

Dermoscopy is a noninvasive tool that was initially used to diagnose cutaneous malignancies, but now has extended its range of application to many inflammatory and pigmentary conditions of the skin [4]. Dermoscopy helps visualize the epidermis, dermo-epidermal junction and papillary dermis, with a variety of morphological structures and patterns aiding in the diagnosis. In this study, we used FotoFinder videodermoscopy to identify the dermoscopic features of PLCA in patients with Fitzpatrick skin types 4 and 5, and correlated these features with histopathological findings.

## Materials and Methods

This was a cross-sectional study of patients attending the dermatology outpatient department at a tertiary care center over 1 year. Patients (aged 15 to 70 years) who had a clinical diagnosis and histopathological confirmation of PLCA were eligible for inclusion in the study. Patients were excluded if they were currently taking medications or had previously taken medications such as topical steroids, topical keratolytic agents or oral antihistamines. Institutional ethical clearance was obtained. Informed consent for performing dermoscopy and skin biopsy and for publishing the results with photographs was obtained from all patients included in the study.

Dermoscopic examination was performed using a Foto- Finder videodermoscope and FotoFinder Universe 2019 software. FotoFinder is a computer-attached, hand-held dermoscope with a range of magnification from 20× to 140×. It provides high magnification and good visualization of skin (epidermis and upper dermis) structures. High quality clinical and dermoscopic images were preserved in the computer and reviewed when necessary. Dermoscopy was performed in the contact-polarized mode with an interface medium when necessary, to avoid reflection due to excessive scales with the help of ultrasound gel [5].

Skin biopsy specimens were stained with hematoxylin and eosin. The presence of amorphous, eosinophilic, acellular material in the papillary dermis was taken as the histopathological diagnostic criteria of amyloidosis.

## Results

**Figure 1:**
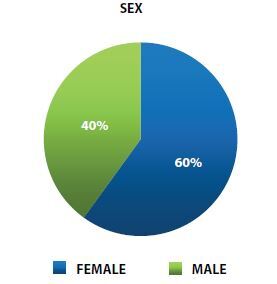
Sex distribution of patients with primary localized cutaneous amyloidosis.

**Figure 2:**
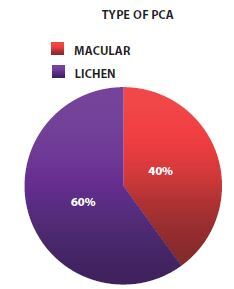
Percentages of patients with lichen and macular amyloidosis in the study.

The study enrolled 30 patients with PLCA, including 18 (60%) females and 12 (40%) males (Figure 1). 21 (70%) patients had a history of pigmented, itchy lesions for more than 1 year. By clinical and histopathological examination, 18 (60%) were diagnosed with LA and 12 (40%) cases with MA (Figure 2).

**Figure 3:**
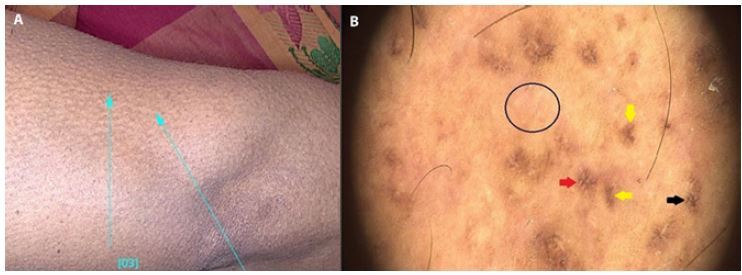
(A) Clinical picture of macular amyloidosis (MA) showing a rippled pattern. (B) FotoFinder dermoscopy image shows a brown central hub and spoke pattern (black arrow), and a white central hub with brown spokes (red arrow). Semicircular hyperpigmented structures in lunar eclipse-shaped structures are seen (yellow arrows). Brown reticular pigment background with diffuse erythematous areas (black circle) are present.

**Figure 4:**
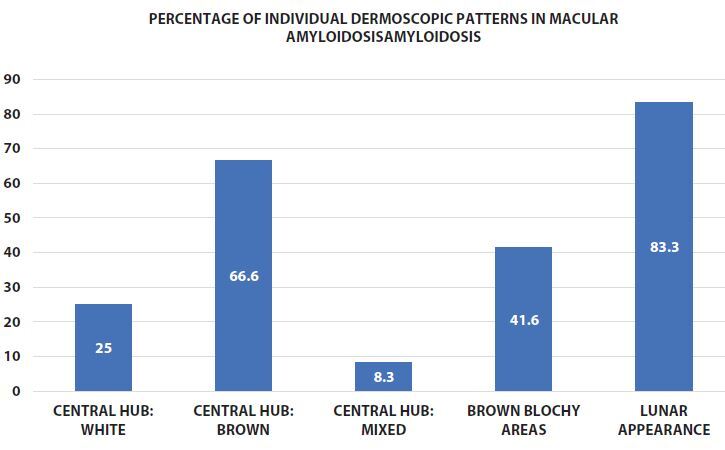
Percentages of individual dermoscopic patterns seen in macular amyloidosis.

**Figure 5:**
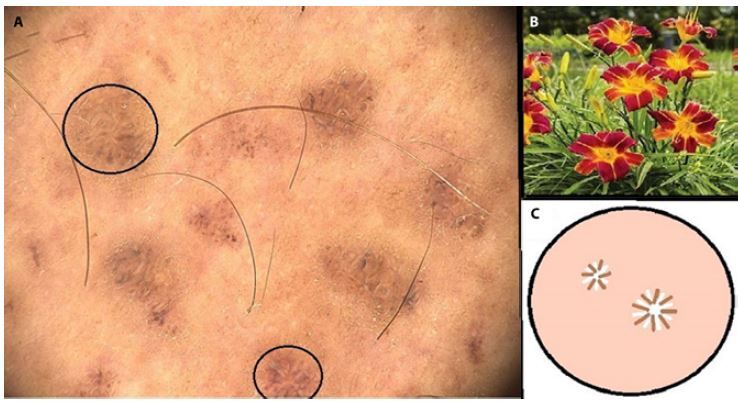
(A) FotoFinder dermoscopy image of MA at 20× magnification showing alternate white and brown striations (black circles). (B) Day lily flower. (C) Schematic of the day lily dermoscopic appearance.

Clinical examination of MA showed hyperpigmented brown macules arranged in a rippled pattern (Figure 3A). Dermoscopy of the lesion revealed the presence of a central hub in all 12 patients (Figure 3B). The frequencies of white, brown and mixed central hubs with brown spokes were 25%, 66.6% and 8.3% respectively (Figure 4). Brown and white hubs appeared as central brown and white dots, respectively, with brown strokes radiating from them. Brown blotchy pigmentation was also seen in 5 patients (41.6%). Semicircular hyperpigmented structures appearing in lunar eclipse-shaped structures (Figure 3B) were also noted in 10 patients (83.3%). These findings were noted on a brown reticular pigment network background. Additionally, 3 patients had an erythematous background. We also noted alternating streaks of brown and white radiating from a white central hub; this new dermoscopic pattern was identified in 2 patients with MA (Figure 5A). It resembles a day lily flower, with alternating bands of reddish-brown and yellow petals (Figure 5B).

**Figure 6:**
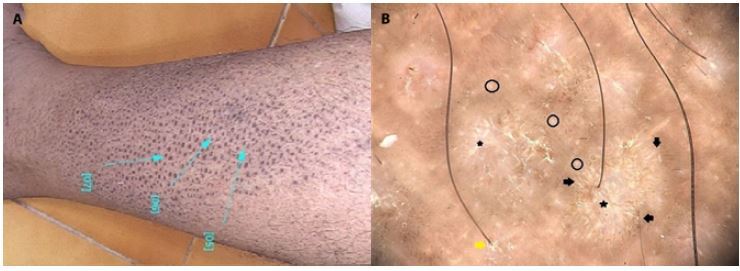
(A) Clinical picture of lichen amyloidosis (LA). (B) FotoFinder dermoscopy image of LA at 20× magnification shows white structureless areas (black stars) over a grayish brown background, with mild diffuse white scales and peripheral alternating white and brown striations (black arrows). White rosettes (black circle) and perifollicular scaling (yellow arrow) are seen.

**Figure 7:**
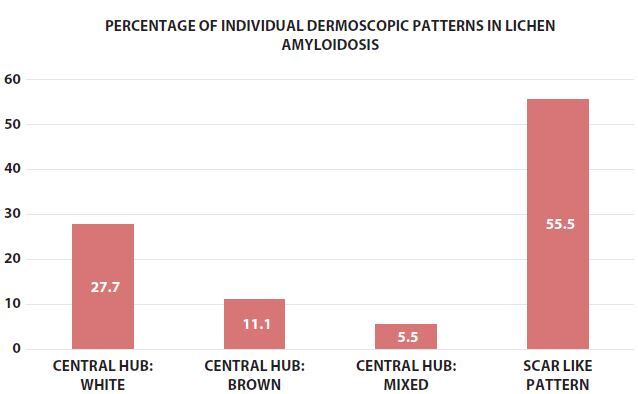
Percentages of individual dermoscopic patterns seen in lichen amyloidosis.

**Figure 8:**
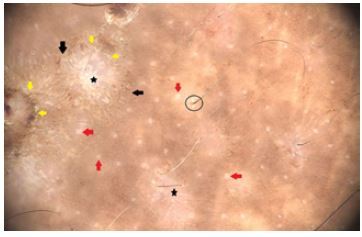
FotoFinder dermoscopic image of LA at 20× magnification shows white structureless areas (black star) over a grayish brown background, white collarette scales (yellow arrows) appearing as volcanic craters, and peripheral alternating white and brown striations (black arrows). White rosettes (red arrows) and perifollicular scaling (black circle) are seen.

**Figure 9:**
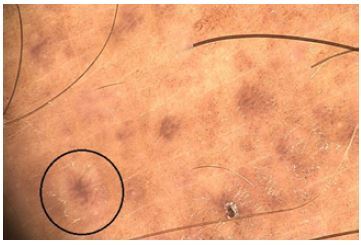
FotoFinder dermoscopy image of LA at 20× magnification shows a central brown hub and spoke pattern with peripheral collarette scaling (black circle).

Clinical examination of LA showed hyperkeratotic grayish to brown papules (Figure 6A) which are non-follicular oriented. Dermoscopy revealed white structureless areas in 10 patients (55.5%) (Figure 7), with a few lesions showing white collarette scaling (Figure 6B) and a few with diffuse white scaling. The white structureless areas appeared to be present on a grayish brown pigment background, which was covered by a peripheral rim of alternating bands of gray and brown pigment striations (Figure 6B and 8) and collarette white scaling (Figures 8 and 9). A central hub was seen in 8 patients (44%), with a frequency of white, brown and mixed patterns of 27.7%, 11.1% and 5.5%, respectively (Figure 7).

We also observed rosettes such as follicular changes under polarized light dermoscopy (Figures 6B and 8) in one patient with LA. Rosettes resemble four-leaf clovers where the 4 leaves represent 4 white, round-to-oval structures attached to the center. Hair follicles were surrounded by white structureless areas, with a few hair follicles having perifollicular scaling (Figures 6B and 8).

**Figure 10:**
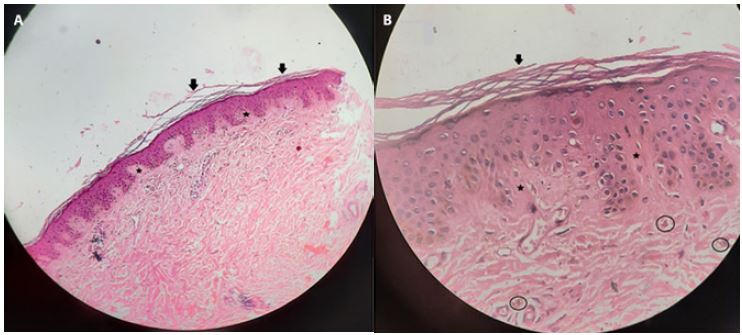
(A) Light microscopy of MA with hematoxylin and eosin staining at 10× magnification shows the presence of basketweave orthokeratosis (black arrow) and eosinophilic deposits in the papillary dermis (black stars). (B) Light microscopy of MA with hematoxylin and eosin staining at 40× magnification shows the presence of basketweave orthokeratosis (black arrow), eosinophilic deposits in the papillary dermis (black stars), and melanophages in the dermis (black circles).

**Figure 11:**
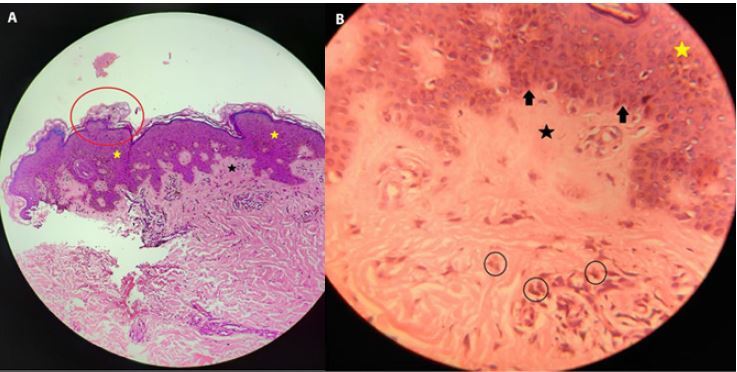
(A) Light microscopy of LA with hematoxylin and eosin staining at 10× magnification shows orthohyperkeratosis (red circle) with acanthosis (yellow stars), broadening of rete ridges and basal hyperpigmentation. Eosinophilic deposits (black star) in the papillary dermis are seen. (B) Light microscopy of LA with hematoxylin and eosin staining at 40× magnification shows acanthosis (yellow stars) with broadening of rete ridges and basal hyperpigmentation (black arrows). Eosinophilic deposits (black star) in the papillary dermis and melanophages (black circle) in the dermis are seen.

Biopsy was performed in all 30 patients. The presence of amorphous, acellular, eosinophilic material deposited in the papillary dermis, indicative of amyloid deposition, was used to diagnose PLCA. Biopsy specimens from MA lesions showed the presence of basketweave orthokeratosis (75%) and hyperkeratosis (33.3%) (Figure 10) with basal hyperpigmentation (100%) and basal vacuolation in the epidermis. Accumulation of amorphous eosinophilic material (100%) in the upper dermis with melanophages was seen. Biopsy specimens of lesions from patients with an erythematous background also showed the presence of a few congested blood vessels in the dermis. A few lesions also showed hyperkeratosis. Biopsy specimens from LA lesions showed orthohyperkeratosis (93.3%) with acanthosis (66.6%), broadening and elongation of rete ridges in the epidermis, and papillomatosis with eosinophilic amorphous material deposition (100%) in the upper dermis (Figure 11). Basal hyperpigmentation along with melanin incontinence (100%) was also observed.

## Discussion

MA and LA are variants of a single pathology, where the amyloid fibrils are derived from keratin intermediate filaments due to epidermal damage and keratinocyte apoptosis [6]. The etiology is either unknown or due to persistent friction. Rokintansky was the first to histopathologically describe amyloidosis in 1842 [7]. Histopathology of the lesions showed amyloid deposits confined to the papillary dermis without involvement of blood and adnexal structures.

MA presents as pruritic, yellowish brown macules with a reticulated or rippled pattern. It is most commonly seen on the upper back, but it can also involve the trunk or extremities. The differential diagnosis of MA includes frictional melanosis and postinflammatory hyperpigmentation, for which a previous histopathological confirmation plays a vital role.

To avoid the need for biopsy in these patients, in this study we tried to corelate the clinical, dermoscopic and histopathological findings. In our study, 12 patients had clinically and histopathologically diagnosed MA. On dermoscopy, we found that 66.6% of patients had brown central hubs, 25% had white central hubs, and 8.3% had both white and brown central hubs (mixed pattern). We found radiating brown streaks from the central hub; this finding was previously reported by Chuang et al.8 who termed it “hub and spoke’ spoke” appearance. We also noted alternate streaks of brown and white radiating from the central hub. This is a new dermoscopic pattern. It is similar to the petals of a day lily flower, with alternating bands of reddish brown and yellow.

Other dermoscopic findings in MA are semicircular, hyperpigmented, lunar eclipse-shaped structures and hyperpigmented blotchy areas seen over a brown reticular pigment background. Some patients also had a diffuse erythematous background.

Brown blotchy pigmentation is the earliest finding of PLCA which on biopsy represents basal hyperpigmentation along with dermal melanin incontinence. With progression of the disease, the brown central hub and spoke pattern was observed on dermoscopy and histopathology showed orthokeratosis. A white central hub corresponded to marked hyperkeratosis, where the excessive stratum corneum prevented the polarized light from scattering and reflected most of the light, thereby preventing visualization of the pigmentation of the basal layer and dermis. Erythematous background is due to congested blood vessels in the dermis.

LA presents as discrete, hyperkeratotic firm red to brownish papules, most commonly situated on the legs, especially the shins and trunk. Usually, the lesions of LA itch severely. Differential diagnosis of LA includes xanthomas, perforating collagenosis, hypertrophic lichen planus and lichen simplex chronicus.

Biopsy of LA showed marked orthohyperkeratosis with acanthosis, which appeared as white structureless areas; these areas seem “scar like” [8] on dermoscopy. This was the commonest finding seen on dermoscopy in 55.5% of patients. The central white scar with peripheral collarette scaling was previously named “volcanic crater” appearance [8]. Although there is increased basal pigmentation, it is not visualized as brown pigmentation on dermoscopy due to the blocking and reflection of light by marked hyperkeratosis and acanthosis. White scaling is due to hyperkeratosis [9]. The peripheral white and brown striations are due to papillomatosis and broadening of rete ridges seen on histopathology. A grayish background is observed on dermoscopy due to the presence of melanin incontinence and melanophages in the dermis.

Rosettes are a new observation made in this study. Rosettes were previously described in squamous cell carcinoma, basal cell carcinoma [10], actinic keratosis and discoid lupus erythematous [11]. Rosette formation is due to the reflection of polarized light through the keratin accumulated at the infundibular level in dilated or blocked infundibula [12].

Orthokeratosis with basal hyperpigmentation and melanin incontinence was a consistent finding in biopsy, and it dermoscopically correlated to a brown central hub. Similarly marked hyperkeratosis was seen consistently with a white central hub. White structureless areas are due to marked orthohyperkeratosis and acanthosis. Peripheral white and brown striations were consistent with papillomatosis and a broadening of rete ridges seen on biopsy.

In this study, along with previously described dermoscopic features, like the central hub and spoke pattern seen in MA and white structureless areas and volcanic crater appearances seen in LA, we found several new patterns: These are the day lily appearance in MA and rosettes in LA. This study nonetheless has several limitations: It was conducted on a small number of patients, and the nodular variant of PLCA was not taken into consideration. Confirmation of the presence of amyloid in the papillary dermis with Congo red staining and viewing under polarized microscopy were also not done in this study.

## Conclusions

This study revealed various dermoscopic findings of MA and LA. It showed consistent correlation between dermoscopy patterns and biopsy findings, which avoid the need for biopsy. The dermoscopic patterns were consistently well corelated with histopathology. With the FotoFinder dermoscope, we had good visualization of the epidermis and the deeper structures and good recognition of various dermoscopic patterns. FotoFinder dermoscopy helps visualize the epidermis and upper dermis and aides in the definite diagnosis of PLCA.
